# Prevalence and related factors of child posttraumatic stress disorder during COVID-19 pandemic: A systematic review and meta-analysis

**DOI:** 10.1192/j.eurpsy.2022.31

**Published:** 2022-06-21

**Authors:** Fan Yang, Jiaxing Wen, Ning Huang, Madelon M. E. Riem, Paul Lodder, Jing Guo

**Affiliations:** 1 School of Public Health, Peking University Health Science Center, Beijing, China; 2 Behavioral Science Institute, Radboud University, Nijmegen, The Netherlands; 3 Clinical Child & Family Studies, Faculty of Behavioral and Movement Sciences, Vrije Universiteit, Amsterdam, The Netherlands; 4 Department of Methodology and Statistics, Tilburg University, Tilburg, The Netherlands

**Keywords:** Child PTSD, COVID-19, meta-analysis, systematic review

## Abstract

**Background:**

The COVID-19 pandemic has drastically impacted many aspects of society and has indirectly produced various psychological consequences. This systematic review aimed to estimate the worldwide prevalence of posttraumatic stress disorder (PTSD) in children due to the COVID-19 pandemic, as well as to identify protective or risk factors contributing to child PTSD.

**Methods:**

We conducted a systematic literature search in the PubMed, ProQuest, PsycINFO, Embase, Web of Science, WanFang, CNKI, and VIP databases. We searched for studies published between January 1, 2020 and May 26, 2021, that reported the prevalence of child PTSD due to the COVID-19 pandemic, as well as factors contributing to child PTSD. Eighteen studies were included in our systematic review, of which 10 studies were included in the meta-analysis.

**Results:**

The estimated prevalence of child PTSD after the COVID-19 outbreak was 28.15% (95% CI: 19.46–36.84%, *I* ^2^ = 99.7%). In subgroup analyses for specific regions the estimated prevalence of post-pandemic child PTSD was 19.61% (95% CI: 11.23–27.98%) in China, 50.8% (95% CI: 34.12–67.49%) in the USA, and 50.08% in Italy (95% CI: 47.32–52.84%).

**Conclusions:**

Factors contributing to child PTSD were categorized into four aspects: personal factors, family factors, social factors and infectious diseases related factors. Based on this, we presented a new framework summarizing the occurrence and influence of the COVID-19 related child PTSD, which may contribute to a better understanding, prevention and development of interventions for child PTSD in forthcoming pandemics.

## Introduction

The impact of the COVID-19 pandemic on our society has been multidimensional [1], affecting various aspects of our lives including psychological consequences such as an increase in posttraumatic stress disorder (PTSD; [2]). PTSD is a common but complex and severe mental disorder which usually occurs after exposure to traumatic events. One study estimated that approximately 10% of the U.S. population will develop symptoms of PTSD at least once during their lifetime [3]. Another study on the mental health impact of the 2014–2016 Ebola epidemics among the general population in affected countries found that 76.4% of the general public showed any symptom of PTSD, 27% met the level of clinical concern for PTSD, and 16% met the level of a probable diagnosis of PTSD [4]. Some studies indicate that children and adolescents might be more prone to adverse impacts caused by traumatic events such as infectious disease pandemics [5–7]. Also, many previous studies have identified PTSD as the most common psychological disorder in adolescents after disasters or trauma [8, 9]. Although the studies related to PTSD are growing, the global prevalence of child PTSD and related risk factors in populations after a pandemic is still unknown.

Several studies have discussed protective or risk factors contributing to child PTSD, including quarantine, pre-existing mental health, coping styles, education, and structure of family [10–12]. Typically, these factors can be divided into three groups: personal factors (i.e., education), family factors (i.e., structure of the family), and social factors (i.e., quarantine). However, during the COVID-19 pandemic, there may also be contributing factors that are directly or indirectly related to pandemic situation. For example, many nonpharmaceutical interventions such as quarantines were a direct consequence of the COVID-19 pandemic [13]. Moreover, school closures and lockdown measures have caused many changes in the lives of both adults and children, such as an increase in screen time [14, 15]. Lastly, during the COVID-19 pandemic, the prevalence of post-COVID-19 diseases, such as multisystem inflammatory syndrome in children and adolescents (MIS-C), has increased as well [16], potentially increasing the risk on childhood PTSD as well [16–18].

Estimating the global burden of child PTSD following pandemics is of vital importance and could aid the development of interventions and management strategies for future pandemics. However, to the best of our knowledge, no systematic review or meta-analysis has reported the pooled prevalence of child PTSD after the COVID-19 pandemic and summarized potential risk factors. Such studies could help guide the public health responses, medical resource allocation, and health policy planning in such worldwide public health emergencies. Here, we conduct a systematic review to estimate the worldwide prevalence and to identify protective or risk factors contributing to child PTSD during the COVID-19 pandemic. Based on our findings we present a new model for child PTSD due to COVID-19.

## Methods

### Search strategy and selection criteria

We performed a systematic review and meta-analysis according to Preferred Reporting Items for Systematic Reviews and Meta-Analysis guidelines (PRISMA; [19]). We systematically searched for literature in the PubMed, ProQuest, PsycINFO, Embase, Web of Science, WanFang, CNKI, and VIP databases to identify studies that were published between January 1, 2020 and May 26, 2021 and reported the prevalence of child PTSD due to COVID-19, as well as risk factors contributing to child PTSD. We searched the databases using the search term: “(children OR adolescent OR (family AND member) OR teenager) AND PTSD AND (infectious OR pandemics OR COVID-19).” Also, we inspected the references lists of included studies and review articles to find additional studies meeting the inclusion criteria.

Two authors (F.Y. and J.W.) independently downloaded and assessed the articles for their eligibility for inclusion. Studies were included if they focused on COVID-19 related child PTSD and met any of following criteria: (a) peer-reviewed articles (articles in Chinese and English were included) that reported the prevalence of child PTSD after COVID-19. We accepted a wide range of PTSD measurement tools, including international diagnostic criteria, actual medical records, and standard questionnaires or instruments; (b) peer-reviewed articles identifying risk factors for post-pandemic PTSD. The following types of articles were excluded: case reports, reviews, and dissertations. If the same sample was used in more than one publication, only the data set with the most comprehensive information was included to avoid data duplication in the meta-analysis.

**Figure 1.**
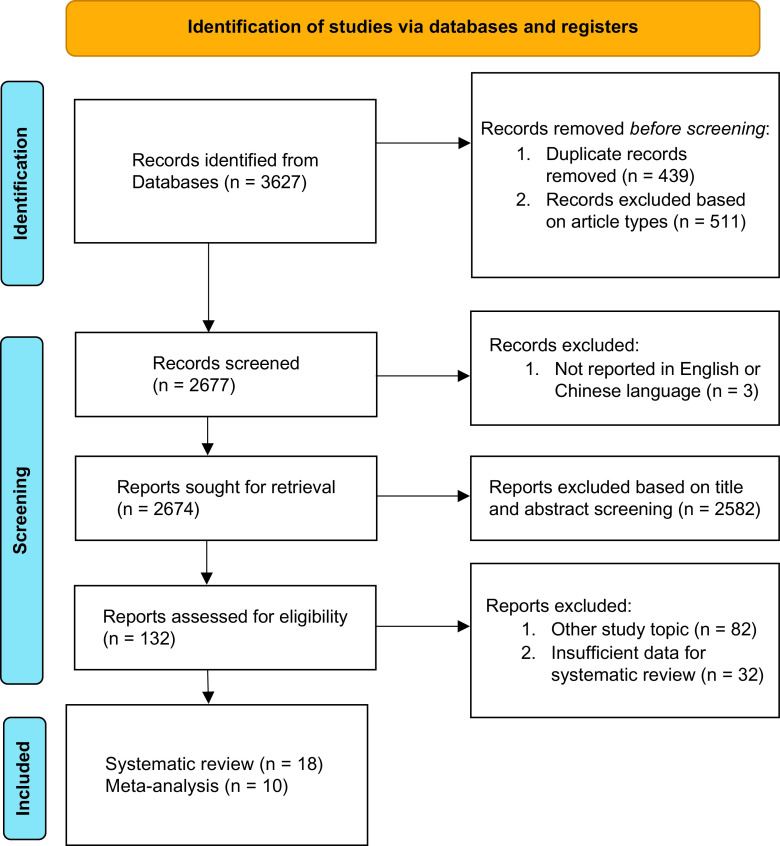
Flow chart of study selection.

Figure 1 shows the flow chart representing the process of identifying eligible studies and reasons for excluding studies. A total of 3627 studies were initially identified, of which 439 were removed as being a duplicate. Another 511 studies were removed because their study type met our exclusion criteria and 3 studies were removed because they did not report their findings in the English or Chinese language. Of the 2,674 studies sought for retrieval, 2582 studies could be excluded based on their title or abstract. Of the 132 screened full texts, 82 studies were excluded because their topic did not meet our inclusion criteria, and 30 studies were excluded because insufficient data were available to include them in the systematic review. In the end, 18 studies (15 written in English and 3 written in Chinese) were included in our systematic review [10–15, 20–31], of which 10 studies were included in our meta-analysis [11, 12, 14, 15, 23–27, 29]. The remaining eight studies could only be included in our systematic review because insufficient data were available for the meta-analysis.

Tables 1 and 2 present the following details for each study included in the meta-analysis (Table 1) and systematic review (Table 2): (a) author(s), (b) publication year, (c) type of research; (d) study region, (e) age of study population, (f) PTSD assessment tool(s), (g) sample size, (h) prevalence of PTSD, and (i) study quality. We used the National Heart, Lung, and Blood Institute (NHLBI) Study Quality Assessment Tools to assess the quality of studies [32]. The data were independently extracted from eligible papers by two authors (F.Y. and J.W.). Also, all discrepancies were resolved by group discussion and consensus.

**Table 1.** Characteristics of the studies included in the meta-analysis.

No.	References	Type of research	Study region	Age (Years, ranges or means)	Assessment tools	Sample size	Population characteristics	Prevalence of PTSD (%)	Study quality
1	Hu et al. [14]	Cross-sectional research	Leshan and Jianyang, Sichuan Province, China	12–18 (Range)	The Children’s Revised Impact of Event Scale (CRIES)	2,090	Senior school students	6.90	Good
2	Krass et al. [23]	Clinical observations	Philadelphia, USA	11–17 (Range)	Clinical diagnosis	19	Laboratory-confirmed COVID-19 hospitalized children	63.00	Fair
3	Li et al. [24]	Cross-sectional research	Hubei, China	8–18 (Range)	The self-reported eight-item Children’s Revised Impact of Event (CRIES-8)	1,172	General children varied in ages	64.51	Good
4	Ma et al. [25]	Cross-sectional research	27 provinces in China	7–15 (Range)	IES-R	668	General children varied in ages	20.70	Good
5	Mensi et al. [26]	Cross-sectional research	Italy	12–18 (Range)	K‐SADS‐PL (DSM‐5) and supplement interview	1,262	General children varied in ages	50.04	Good
6	Murata et al. [27]	Cross-sectional research	United States	13–18 (Range)	PC-PTSD-5(the Primary Care PTSD Screen for DSM‐5)	583	Children lost someone due to COVID-19	45.00	Good
7	Shek et al. [11]	Cross-sectional research	Chengdu, China	13.15 (Mean)	The Children’s Revised Impact of Event Scale (CRIES-13)	4,981	General children varied in ages	10.40	Good
8	Yue et al. [15]	Cross-sectional research	Jiangsu, China	10.56 (Mean)	PTSD Checklist for DSM-5 (PCL-5)	1,360	General children varied in ages	3.16	Fair
9	Zhang et al. [12]	Cross-sectional research	Sichuan, China	Not reported	PCL-C	4,225	Middle school students	10.60	Fair
10	Zhang et al. [12]	Cross-sectional research	Guangzhou, Guang Dong, China	13.93 (Mean)	IES-R	1,025	Middle school students	21.70	Good

**Table 2.** Characteristics of the studies included in the systematic review in addition to those reported in Table 1.

References	Type of research	Region of research	Age (Years, ranges or means)	Assessment tools	Sample size	Prevalence of PTSD	Reason for excluding	Study quality
Conti et al. [20]	Longitudinal study	Italy	1.5–18 (Range)	CBCL	141	Not reported	Lack of essential data	Good
Guo et al. [21]	Cross-sectional research	Wu Han, China	11–18 (Range)	PCL-5	6,196	8.00%	Different topic	Good
Jiang et al. [22]	Cross-sectional research	China	25.76 (Mean)	PCL-5	338	3.50%	Adult sample	Good
Liu et al. [10]	Cross-sectional research	50 U.S. states	18–30 (Range)	PCL-C	898	31.80%	Adult sample	Fair
Lu [13]	Commentary	Not reported	Not reported	Not reported	Not reported	Not reported	Lack of essential data	Fair
Sharpe et al. [28]	Cross-sectional research	Sierra Leone and Zambia	12–25 (Range)	/	468	/	Lack of essential data	Good
Zhu et al. [30]	Cross-sectional research	China	Not reported	PCL-C	343	Not reported	Lack of essential data	Fair
Zolnikov et al. [31]	Commentary	Not reported	Not reported	Not reported	Not reported	Not reported	Lack of essential data	Fair

### Data analysis

All analyses were conducted by *R* 4.1.0. and *R*-package “meta” [33, 34]. To accommodate for potential heterogeneity in the included effect sizes due to different study populations and assessment methods, a random effect was used to estimate the pooled prevalence. As opposed to a fixed effects model it does not assume that the true prevalence is the same in all included studies and therefore introduces another source of uncertainty to the overall prevalence estimate, increasing the width of the confidence interval for the overall prevalence estimate. Also, we used an *I* ^2^ statistic estimate of ≥50% as an indicator of considerable statistical heterogeneity. To explore potential sources of this heterogeneity, subgroups and meta-regression analyses were conducted based on study regions. Egger’s test and Begg’s test were performed to assess publication bias and small-study effects and funnel plots were used to visualize the prevalence estimates against the precision of their estimate. Moreover, sensitivity analyses were conducted to estimate the influence of individual studies on the pooled estimates by excluding each of the studies from the pooled estimate.

## Results

### Post-pandemic PTSD prevalence

Of the 10 studies that reported prevalence data, three major populations were analyzed and described: seven studies in Chinese populations (total *n* = 15,521), two studies in American populations (total *n* = 602) and one study in the Italian population (total *n* = 1,262). All these studies focused on COVID-19 related child PTSD. A total of nine studies (90%) used a questionnaire to identify the PTSD, whereas only one study (10%) made a PTSD diagnosis by professional mental health workers.

**Figure 2.**
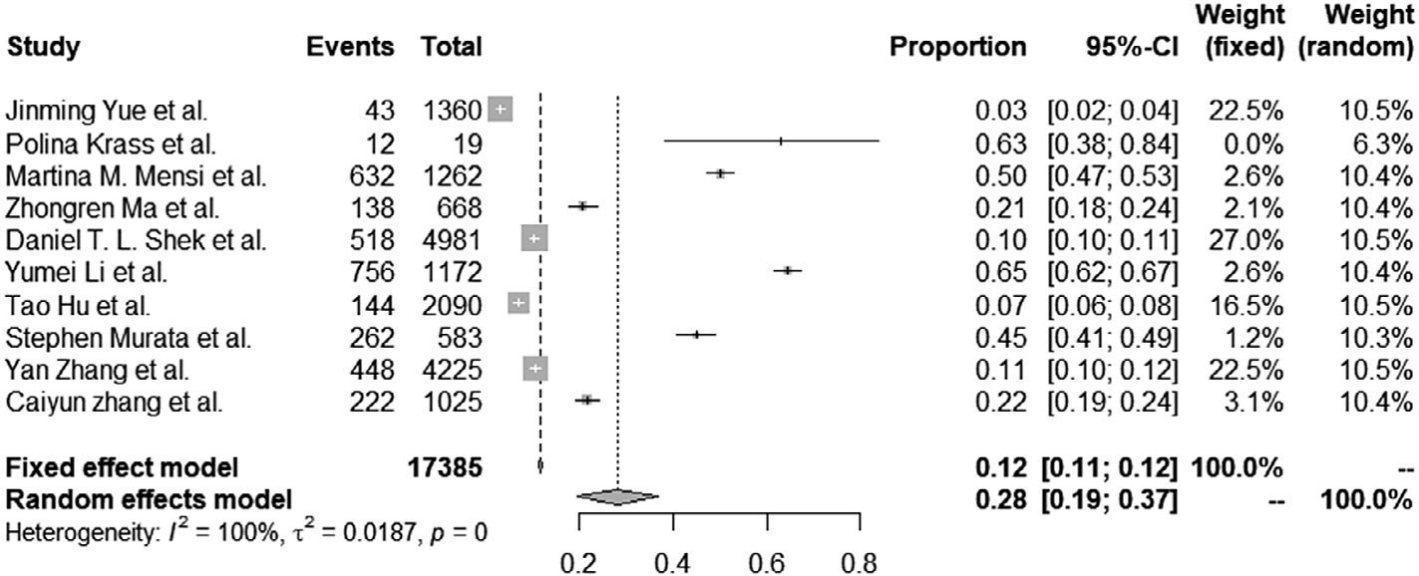
Estimated prevalence of post-pandemic child PTSD across all 10 studies included in the meta-analysis. The dashed and dotted vertical lines represent the overall prevalence estimates according to the fixed and random effects model, respectively.

**Figure 3.**
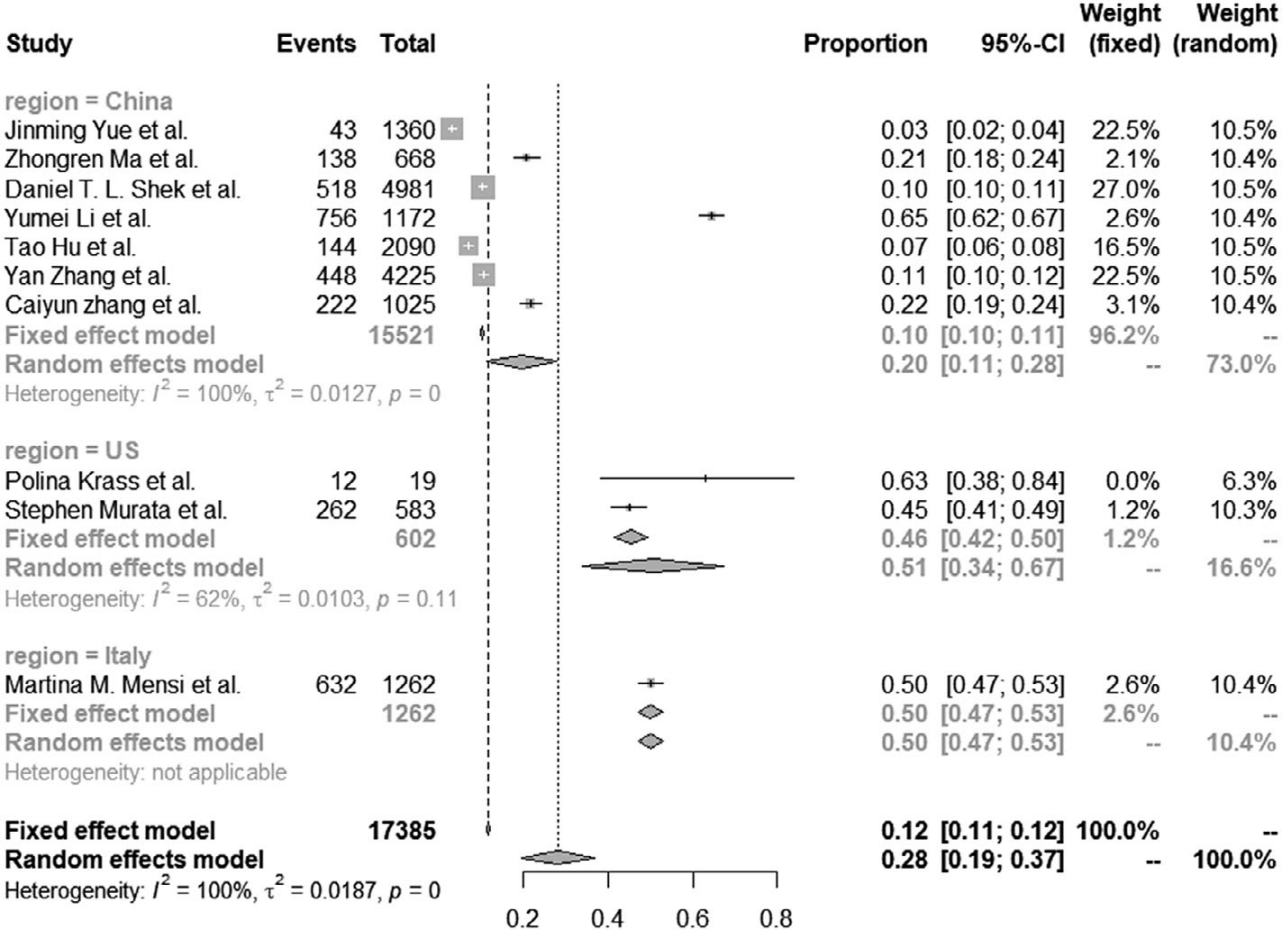
Subgroup analysis of post-pandemic child PTSD prevalence across three different study regions. The dashed and dotted vertical lines represent the overall prevalence estimates according to the fixed and random effects model, respectively.

The estimated prevalence of child PTSD after the outbreak was 28.15% (95% CI: 19.46–36.84%, *I* ^2^ = 99.7%, see Figure 2). We performed subgroup analyses according to the region of study populations (see Figure 3) and estimated that the prevalence of post-pandemic child PTSD was 19.61% (95% CI: 11.23–27.98%) in the Chinese population, 50.8% (95% CI: 34.12–67.49%) in the American population and 50.08% in the Italian population (95% CI: 47.32–52.84%). A meta-regression indicated significant differences between study regions in the estimated child PTSD prevalence (*p* < 0.001).

**Figure 4.**
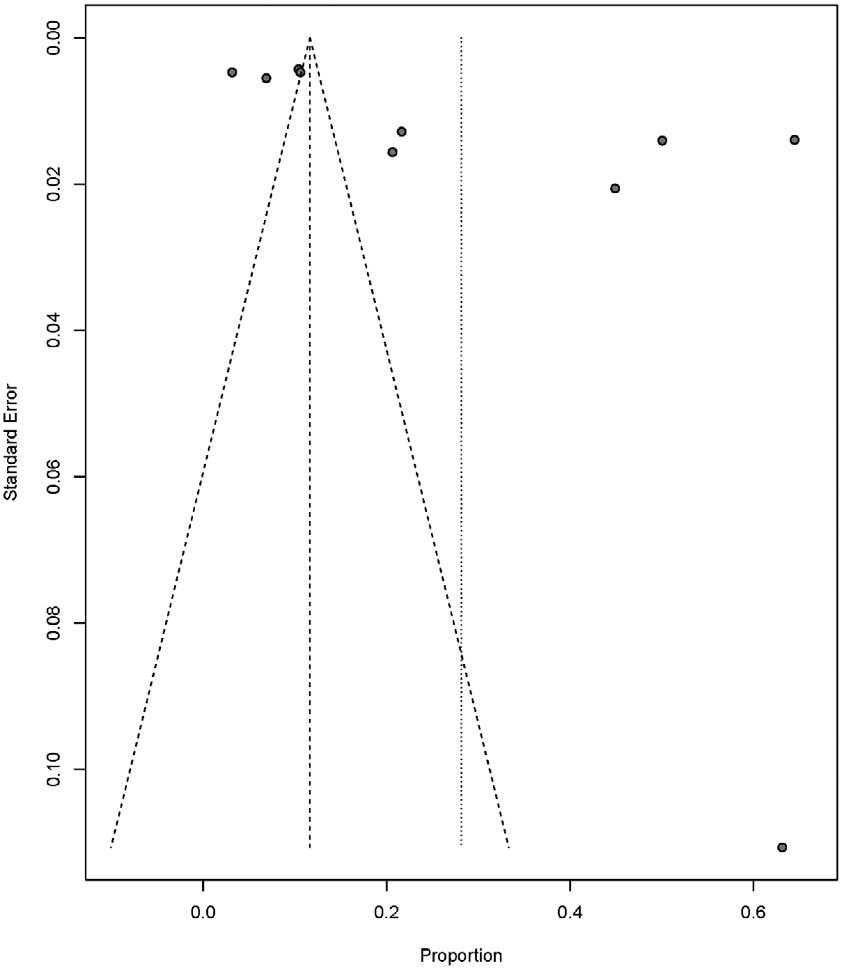
Funnel plot. Begg’s test indicated no apparent publication bias while Egger’s tests indicated there was significant publication bias (*p*-value of Begg’s is greater than 0.05 while Egger’s test’s was lower than 0.05).

Begg’s test indicated no apparent publication bias (*z* = 1.34, *p* = 0.18 > 0.05) while Egger’s tests indicated significant publication bias in our study (*t* = 2.93, *p* = 0.019 < 0.05) and visualization of bias was presented as a funnel plot (Figure 4). This difference in conclusion between the two tests might be caused by differences in statistical power due to the limited number of studies included in the analysis. Also, the funnel plot and tests for publication bias assume homogeneous effects. This assumption may be violated in the context of our analysis because of the heterogeneous study populations and PTSD assessment methods. To investigate whether individual studies have a major impact on our conclusions, we conducted leave-one-out sensitivity analyses that excluded each individual study before estimating PTSD prevalence. The results showed that after each excluded study, the meta-analysis still resulted in similar estimates of PTSD prevalence, suggesting that no single study included in the meta-analysis was likely to have an inordinate impact on the reported prevalence estimates. Therefore, the publication bias might be due to the limited number of studies or due to differences between studies in population or methodological characteristics.

### Potential influential factors for post-pandemic child PTSD

Eighteen eligible studies discussed risk factors for child PTSD related to infectious diseases. However, due to the limited number of studies on each factor and the lack of required data on these factors within a number of studies, it was not possible to conduct a meta-analysis. Therefore, we describe discuss these risk factors of child PTSD in a systematic review. We classified each risk factor into one of four categories: (a) personal and family characteristics, (b) infectious diseases related factors, (c) internal and external support system (‘external system’ refers to support from outside the individual [e.g., government], while ‘internal system’ refers to supporting individual perspective [e.g., resilience]), and (d) psychological and behavioral changes [10–15, 20–31].

With regard to personal and family characteristics, the included studies show that males with married parents, higher education and household income were at lower risk of developing PTSD [11, 25, 27–30], while factors such as negative experience (e.g., problematic youth development, maltreatment before pandemics and life time history of suicidal ideation), poor health status before the pandemic (e.g., disability or pre-existing mental health problems) and female gender were related to a higher risk on post-pandemic PTSD [10, 11, 14, 15, 21, 27, 28]. Another risk factor is the father’s occupation (e.g., a child with a father occupied as farmer was more at risk of PTSD than when the father was occupied as a businessman; [25]). Also, the primary caregiver (i.e., a child living with parents was less likely to have PTSD symptoms compared to a child living with grandparents or other caregivers) and location of residence (i.e., a child living in Zhejiang province was less likely to have PTSD symptoms compared to a child living in Liaoning province due to differences in policies to handle the pandemic) could impact the prevalence of PTSD as well [25].

With regard to COVID-19 related factors that increase the risk of developing child PTSD, we can distinguish between factors directly related to the virus itself (e.g., perceived threat of COVID-19, health worries, or hospitalization), and factors related to nonpharmaceutical interventions to handle the pandemic (e.g., lockdown-related stressors, quarantine, school closures, and financial hardship) [11, 13, 14, 20, 21, 27].

With respect to the internal system, studies have shown that resilience and positive coping could decrease the possibility of developing PTSD after a potentially traumatic occurrence related to a pandemic [12]. As for other psychological and behavioral changes, several studies have found that increased loneliness, anxiety and depressive symptoms [24, 27, 30], as well as long screen time, and daytime sleepiness could increase the risk of developing PTSD [14, 15, 21].

## Discussion

To our knowledge, this is the first systematic review and meta-analysis that specifically aimed to estimate the prevalence of child PTSD after the COVID-19. There are several previously published meta-analyses and systematic reviews, though with a focus different from the current study. For instance, Yuan et al. [35] discussed the prevalence of PTSD after infectious disease in the general population, while Beaglehole et al. [36] reviewed the occurrence of PTSD after natural disasters like floods, and other significant traumas (i.e., the World Trade Center disaster in 2001; [37]). A recent study [35] indicated a post-pandemic estimated pooled PTSD prevalence of 23% in the general population, which is somewhat lower than our estimate (28.15%) in child populations. Our results add to previous studies and indicate that compared to the general population, children could be at a higher risk of developing PTSD after an infectious diseases pandemic.

Also, we compared the child PTSD prevalence estimates across different study regions. The prevalence estimates in the Chinese population was significantly lower than the Italian and American population estimates. On the one hand, these differences might be due to the different governmental policies to handle the COVID-19 pandemic, indirectly impacting the prevalence of PTSD. On the other hand, cultures differences could also have influenced these estimates, such as differences in the exposure to, appraisal of and response to traumatizing events [38]. Also, cultural beliefs determine trauma vulnerability and resilience as well [39]. Notably, differences between the included studies in the study population may have produced differences between regions in the PTSD prevalence estimates (see Table 1). The studies in the U.S. sampled “laboratory-confirmed COVID-19 hospitalized children” and “children who lost someone due to the COVID-19.” Such children might be at increased risk compared with other populations (i.e., healthy children from the general population). This may have resulted in the higher prevalence estimates in the U.S. than in other regions.

In this study, we reviewed studies that examined risk factors for child PTSD and categorized the large amounts of related factors for post pandemics PTSD into four categories: (a) personal and family characteristics, (b) infectious diseases related factors, (c) internal and external support system, and (d) psychological and behavioral changes. These findings could be potentially informing intervention or treatment of infectious diseases pandemics related child PTSD and future pandemics.

Personal characteristics accounted for most of the factors. Previous studies suggest that female gender is a significant risk factor for developing PTSD [40–43]. The results of our systematic review are in line with these previous findings as the reviewed studies show that girls are at increased risk for post-pandemic PTSD [14, 15]. Moreover, the most commonly identified socioeconomic factor contributing to risk for PTSD was education [11, 25, 29, 30]. On the one hand, higher education is related to lower risk for PTSD [11, 25, 30], which has also been found in previous studies [12, 22, 44, 45]. On the other hand, children of specific grades (i.e., the third grade of high school) have been found to be at increased risk for PTSD, which could be a consequence of higher pressure and anxiety for study outcomes like entrance examination [29]. As for the negative or positive experience before the pandemic, a positive youth development could decrease the risk of developing PTSD as well. Such positive development could make children more concerned about their family, thus the relationship among family members could be more positive, which may benefit their mental health status [46]. On the contrary, pre-pandemic experiences of maltreatment, a history of suicidal ideation and boarding school may interfere with child development and could increase the risk of developing PTSD [21, 25, 27]. Also, children with pre-existing mental health problems (i.e., anxiety) and disability were at increased risk for PTSD [10, 28].

Family characteristics included family economic status, such as household income and father’s occupation, and household composition such as single parenthood, primary caregiver (i.e., living with parents or living with grandparents) and location of residence [12, 25, 27]. These results were in line with previous studies. For example, economic disadvantages are significantly associated with children’s mental health [47, 48] and the father’s occupation contributed to the prevalence of PTSD. More specifically, a father occupied as a farmer was the highest risk factor while being occupied as a businessman was the most protective factor [25]. In addition, children who are raised by a single parent showed worse mental health than children living with both parents family [49], which was inline with our findings as well [29]. Moreover, children living with parents had a lower risk of developing PTSD than those who do not live with parents [25], which is consistent with former studies [50,51]. Notably, a child’s location of residence had an influence on PTSD and this might be because different regions have employed different strategies to handle infectious diseases and may also have different levels of economic development, thereby differentially impacting children mental health [52].

Based on previous research [10], infectious diseases related factors can be divided into two groups: virus-related stressors such as hospitalization, the perceived threat of COVID-19 and health worries and the indirect consequences of interventions to handle the pandemic, such as lockdown-related stressors, quarantine, school closures, and financial hardship [11, 13, 14, 20, 21, 27]. On the one hand, a pandemic could result in high concerns about the virus itself, and the high infectivity, mortality rates and transmission speed of the Sars-CoV-2 virus could induce a high perceived threat of the virus and health worries about COVID-19. These factors could increase the risk of developing PTSD, which is consistent with previous studies [53–55]. Although lockdown measures can limit the spread of the virus, they can also indirectly increase the risk of PTSD due to economic depression and social isolation [13, 15].

Both the virus-related stressors and lockdown-related stressors could disrupt the internal and external system. With respect to the external system, pandemic diseases have a wide impact on many aspects of a society [56], such as the interruption of social support systems and an unstable social environment, which could increase stress and contribute to negative psychological responses [24, 27, 30] and new social public problems. Regarding the internal system, some studies have shown that quarantine could regulate and protect family functioning in time of crisis and inhibit or interrupt the functions of individuals, peers, family and community resilience [57–59]. Higher risk perception could reduce psychological well-being via increasing problem-focused coping [60]. When positive coping and resilience is reduced or even absent in the internal system, children may be at greater risk of PTSD [12].

Moreover, it should be noted that changes in the internal or external system and stressors related to virus and lockdown can trigger other negative behavioral and psychological changes in children. Specifically, these factors strengthened the negative feelings, such as loneliness [27], worries about diseases [11], and increased the possibility of unhealthy behaviors (i.e., problematic smartphone use and long screen time; [14, 15]). Our review highlights the multidimensional impact of pandemic diseases and a complete assessment requires taking into account all of these factors and how they may influence each other.

**Figure 5.**
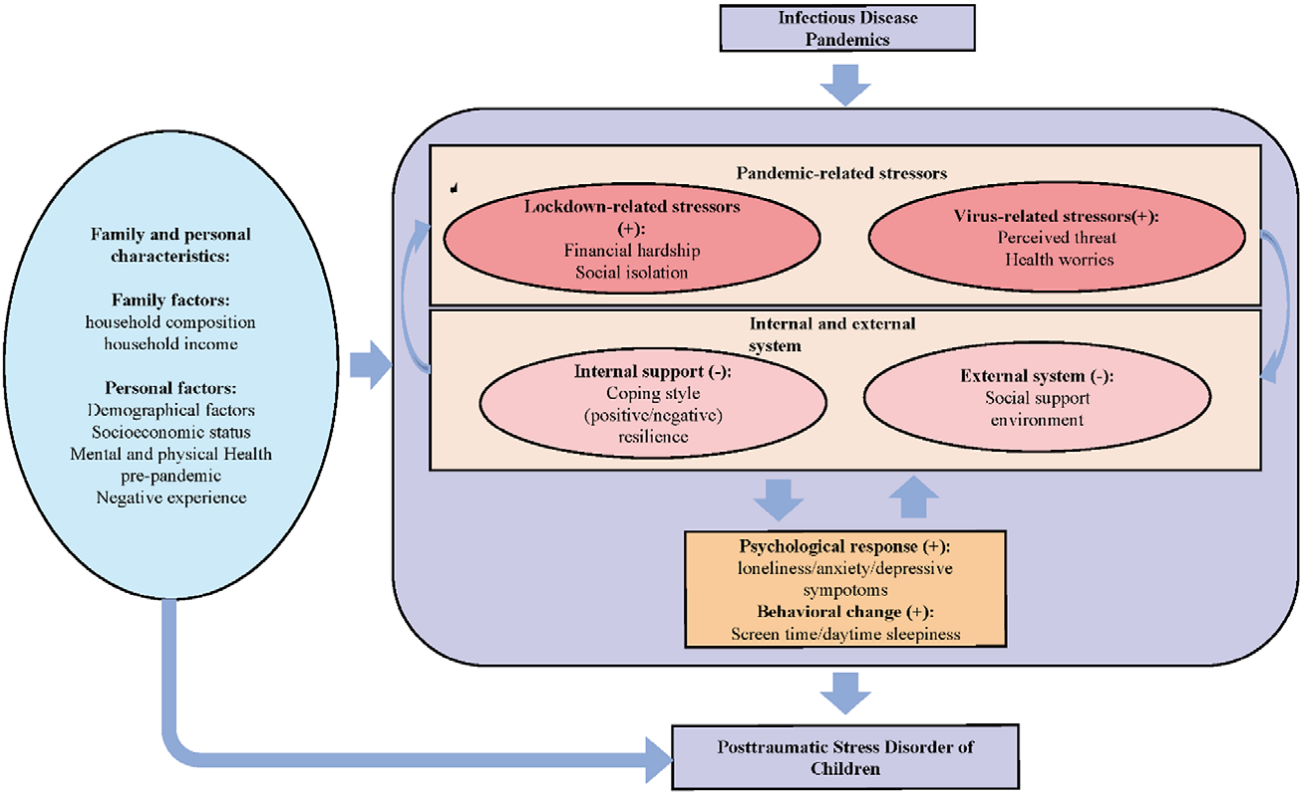
A new framework for the occurrence and contributing factors of post-pandemic child PTSD.

Based on the preceding discussion, we have formulated a new framework to summarize the influence of the pandemics and related risk factors on the development of PTSD. Figure 5 visualizes our framework. A pandemic not only produces virus-specific stressors, but also internal and external system changes, psychological and behavioral responses, and all these factors could be affected by personal and family characteristics in this framework. Together, these characteristics are vulnerability factors for PTSD. It is important to note that pandemic-related stressors and internal and external systems can mutually influence each other. Among them, pandemic-related stressors could result in a negative psychological or behavioral response, and hence an increased risk of PTSD. On the contrary, a stable internal and external system, meaning sufficient support, resources, and a stable environment, could reduce negative emotions and unhealthy behaviors, and thereby prevent the occurrence of PTSD. Each of the four identified categories of factors could affect the development of PTSD among children during a pandemic. Of note, some personal responses could impact societal factors as well (i.e., negative psychological and behavioral response at individual level might raise negative social sentiments and produce a unstable social environment, thus strengthening the effect of the stressors). Therefore, it is important that future research studies the potential pathways by which pandemics can increase prevalence of PTSD and to adapt interventions of PTSD more precisely.

## Limitations and Contributions

This study has several potential limitations. First, the number of included studies was relatively small unpublished studies or other gray literature were not included in the meta-analysis, which may have affected the prevalence estimates. Second, we discovered substantial heterogeneity in the estimates of PTSD prevalence across studies, which might be explained by the limited number of studies in our meta-analysis or the large differences between studies in for instance the type of child population. Third, the diversity of the scales might influence the results to some extent, with self-report scales likely resulting in different prevalence estimates than clinical diagnoses. Fourth, the sampled participants in the included studies were living in a small selection of countries, which limits the generalizability of our study to those countries. Fifth, our findings could not be compared to a baseline child PTSD rate in the advent of the COVID-19 pandemic. A possible reason may be that PTSD is often related to an unforeseen disaster, often resulting in data collection after the traumatic event. Furthermore, child PTSD received less attention compared to PTSD in the general population, which in part motivated the current research. Another limitation is that there was insufficient data to investigate subgroup comparisons that could be stratified by other variables associated with PTSD, such as race, sex, age, and pre-existing mental health diagnosis [61]. Therefore, future research could use individual patient data meta-analysis to pool the raw data of eligible studies and investigate which individual characteristics increase the risk of developing PTSD after COVID-19 or another pandemic.

Although there are several limitations, our study still contributes to the literature on the impact of pandemics on mental health. First, to the best of our knowledge this is the first systematic review estimating the overall prevalence of child PTSD due to the COVID-19 pandemic. Furthermore, we studied risk factors of developing PTSD related to infectious diseases in different child populations across three countries and categorized the identified (protective or risk) factors in four main categories. Also, the combined prevalence of PTSD in children after infectious disease pandemics was 28.15% and exceeded the combined prevalence of PTSD of all individuals according to Kai et al.’s work [35]. Also, our results indicate that PTSD is a significant public health problem and is associated with a large number of risk factors. We recommend policy makers to take risk and protective factors into consideration when designing policies to reduce the personal or societal impact of PTSD. Besides, we presented a new framework to summarize the occurrence and influence of the pandemic-related child PTSD, which may contribute to a better understanding, prevention and development of interventions for child PTSD during pandemics. Early prevention and intervention should be implemented specifically and comprehensively according to the currently identified influential factors, in particular in vulnerable populations such as low-income populations and girls.

## Conclusions

In conclusion, the combined prevalence of PTSD in children after the COVID-19 was 28.15%. American populations showed the highest prevalence, followed by Italy and China. These findings indicate that during and after infectious disease pandemics, post-pandemic child PTSD might be a severe worldwide public health problem that should receive more attention. Also, a large number of factors influence the prevalence and occurrence of post-pandemic child PTSD. Therefore, public health strategies should take these factors into consideration, especially for vulnerable populations (i.e., children who live with single parents and children with quarantine experience). In addition, we presented a new framework summarizing our findings, contributing to a better understanding of post-pandemic child PTSD and informing prevention and intervention. Future research could use longitudinal designs with longer follow-up times to study how infectious diseases such as COVID-19 impact the course of PTSD in children across time.

## Data Availability

Data that supports the findings of this study are available from the corresponding author upon request.
